# Supplementary material for: The Development and Assessment of a Unique Disulfidptosis-Associated lncRNA Profile for Immune Microenvironment Prediction and Personalized Therapy in Gastric Adenocarcinoma
Source: Biomedicines. 2025 May 19;13(5):1224. doi: 10.3390/biomedicines13051224 (PMC12109475; doi:10.3390/biomedicines13051224)
Supplement: Supplementary file 1 [file biomedicines-13-01224-s001.zip › Supplementary_Figure_S2_.pdf]

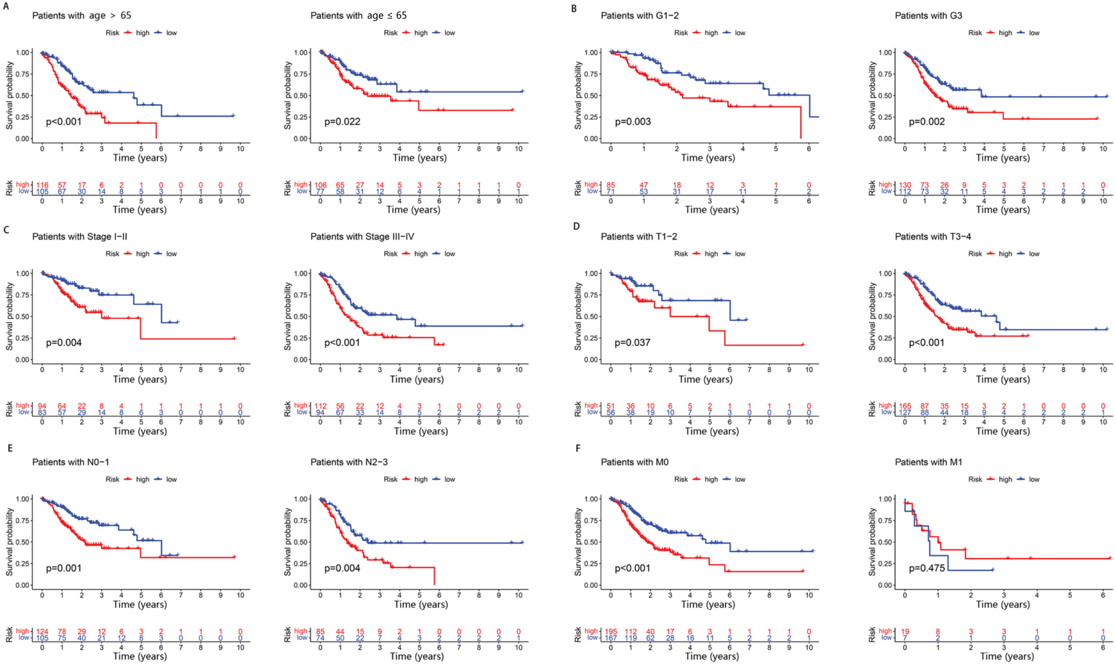

A
Patients with age > 65
p<0.001
Patients with age ≤ 65
p=0.022
B
Patients with G1−2
p=0.003
Patients with G3
p=0.002
C
Patients with Stage I−II
p=0.004
Patients with Stage III−IV
p<0.001
D
Patients with T1−2
p=0.037
Patients with T3−4
p<0.001
E
Patients with N0−1
p=0.001
Patients with N2−3
p=0.004
F
Patients with M0
p<0.001
Patients with M1
p=0.475
Risk high low
Survival probability
Time (years)
