# Supplementary figures and images for: The Development and Assessment of a Unique Disulfidptosis-Associated lncRNA Profile for Immune Microenvironment Prediction and Personalized Therapy in Gastric Adenocarcinoma
Source: Biomedicines. 2025 May 19;13(5):1224. doi: 10.3390/biomedicines13051224 (PMC12109475; doi:10.3390/biomedicines13051224)

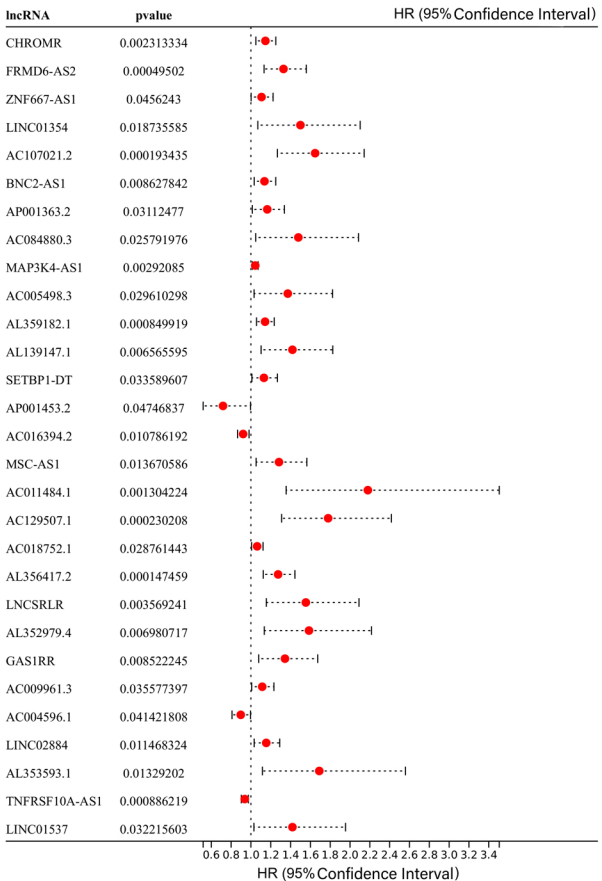

Supplement: Supplementary file 1 [file biomedicines-13-01224-s001.zip › Supplementary_Figure_S1_.pdf]

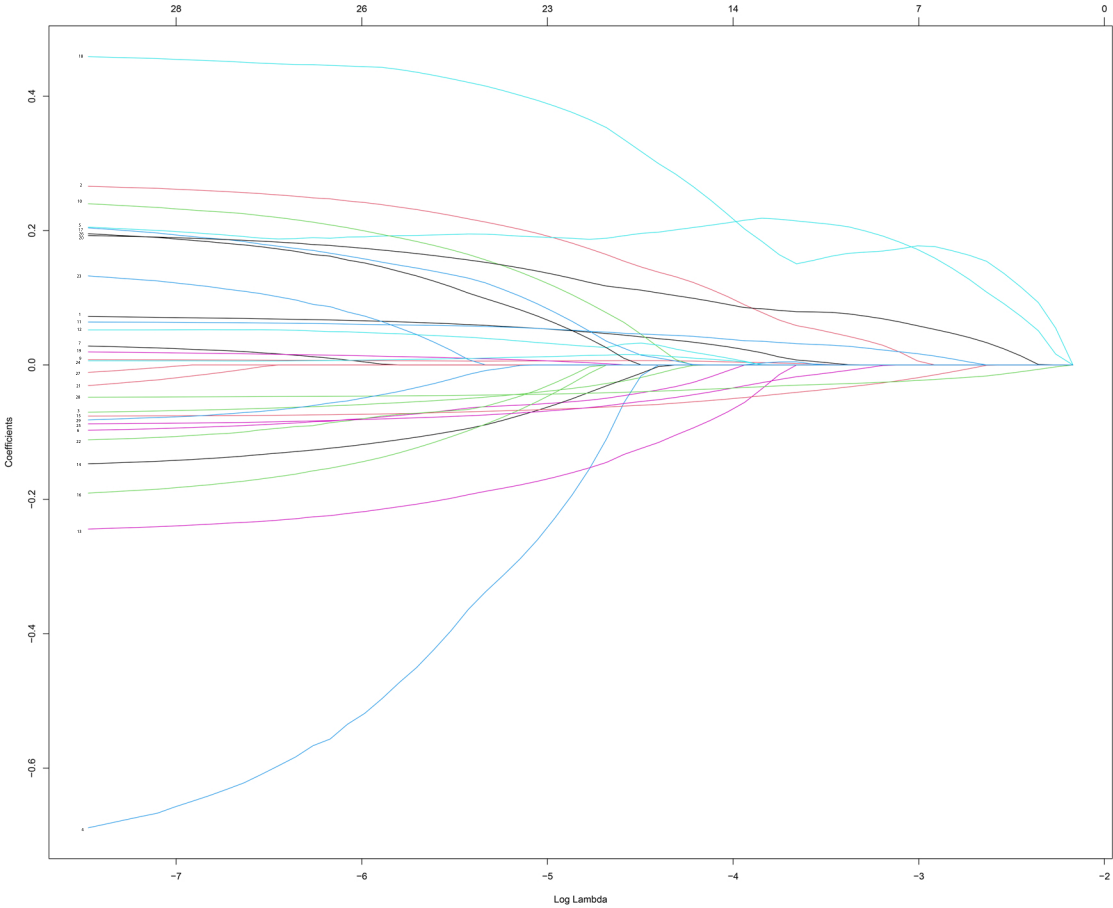

Supplement: Supplementary file 1 [file biomedicines-13-01224-s001.zip › Supplementary_Figure_S3_.pdf]
